# Dibut­yl{*N*′-[1-(5-chloro-2-oxidophenyl-κ*O*)ethyl­idene]-3-hy­droxy-2-naphtho­hydrazidato-κ^2^ *N*′,*O* ^2^}tin(IV)

**DOI:** 10.1107/S1600536810021896

**Published:** 2010-06-16

**Authors:** See Mun Lee, Hapipah Mohd Ali, Kong Mun Lo

**Affiliations:** a Department of Chemistry, University of Malaya, 50603 Kuala Lumpur, Malaysia

## Abstract

The five-coordinate Sn^IV^ atoms in the two crystallographically independent mol­ecules of the title compound, [Sn(C_4_H_9_)_2_(C_19_H_13_ClN_2_O_3_)], are in distorted *cis*-C_2_NO_2_Sn trigonal-bipyramidal coordination environments. The tridentate dianion of the Schiff base, *N*′-[1-(5-chloro-2-oxidophen­yl)ethyl­idene]-3-hy­droxy-2-naphtho­hydrazide, displays inter­molecular O—H⋯N hydrogen bonding, which stabilizes the overall compound.

## Related literature

For a related structure, see: Lee *et al.* (2009 ▶). For the specific biological activity of metal complexes with hydrazone ligands, see: Bernhardt *et al.* (2006 ▶); Ainscough *et al.* (1999 ▶); Mohd Ali *et al.* (2004 ▶).
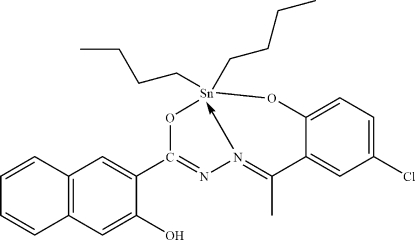


## Experimental

### 

#### Crystal data

[Sn(C_4_H_9_)_2_(C_19_H_13_ClN_2_O_3_)]
*M* *_r_* = 585.67
Monoclinic, 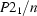

*a* = 24.8256 (13) Å
*b* = 7.1994 (4) Å
*c* = 28.3649 (15) Å
β = 96.376 (1)°
*V* = 5038.3 (5) Å^3^
*Z* = 8
Mo *K*α radiation
μ = 1.15 mm^−1^
*T* = 100 K
0.25 × 0.25 × 0.15 mm

#### Data collection

Bruker APEXII CCD area-detector diffractometer
Absorption correction: multi-scan (*SADABS*; Bruker, 2009 ▶) *T*_min_ = 0.762, *T*_max_ = 0.846
37459 measured reflections
8881 independent reflections
8369 reflections with *I* > 2σ(*I*)
*R*_int_ = 0.032

#### Refinement

*R*[*F* ^2^ > 2σ(*F* ^2^)] = 0.050
*wR*(*F* ^2^) = 0.101
*S* = 1.32
8881 reflections
621 parameters
H-atom parameters constrained
Δρ_max_ = 0.82 e Å^−3^
Δρ_min_ = −1.50 e Å^−3^

### 

Data collection: *APEX2* (Bruker, 2009 ▶); cell refinement: *SAINT* (Bruker, 2009 ▶); data reduction: *SAINT*; program(s) used to solve structure: *SHELXS97* (Sheldrick, 2008 ▶); program(s) used to refine structure: *SHELXL97* (Sheldrick, 2008 ▶); molecular graphics: *X-SEED* (Barbour, 2001 ▶); software used to prepare material for publication: *publCIF* (Westrip, 2010 ▶).

## Supplementary Material

Crystal structure: contains datablocks I, global. DOI: 10.1107/S1600536810021896/hg2693sup1.cif

Structure factors: contains datablocks I. DOI: 10.1107/S1600536810021896/hg2693Isup2.hkl

Additional supplementary materials:  crystallographic information; 3D view; checkCIF report

## Figures and Tables

**Table 1:** Hydrogen-bond geometry (Å, °)

*D*—H⋯*A*	*D*—H	H⋯*A*	*D*⋯*A*	*D*—H⋯*A*
O3—H3*A*⋯N2	0.84	1.85	2.602 (5)	147
O6—H6*A*⋯N4	0.84	1.88	2.617 (5)	146

**Fig. 1.**
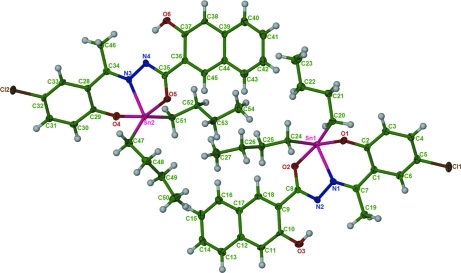
The molecular structure of {N'-[1-(5-chloro-2-oxidophenyl-κO)ethylidene]-3-hydroxy-2- naphthohydrazidato-κ2N',O}dibutyltin(IV) showing 50% probability displacement ellipsoids and the atom numbering. Hydrogen atoms are drawn as spheres of arbitrary radius.

## supplementary crystallographic information

### Comment

Schiff bases derived from substituted salicylaldehydes have been widely used as polydentate ligands in the preparation of metal complexes. The metal complexes of these hydrazones with substituted salicylaldehydes are known to possess potential biological activities such as antifungal, anticancer and many others [Bernhardt *et al.* (2006), Ainscough *et al.* (1999), Mohd Ali *et al.* (2004)]. We have earlier reported the synthesis and molecular structure of a diphenyltin complex of the Schiff base derived from the reaction of 3-hydroxy-2-naphthoic hydrazide with 5-chlorobenzaldehyde [Lee *et al.* (2009)]. The crystal structure of this complex consists of discrete molecules in which the tin atom is *O,N, O'*-chelated by the deprotonated Schiff base ligand. As an extension of our work in structural characterization of organotin with hydrazones, we report here the molecular structure of a dibutyltin complex of a Schiff base derived from the reaction of 3-hydroxy-2-naphthoic hydrazide with 5-chloro-2-hydroxyacetophenone. The unit cell of the title complex consists of two crystallographically independent molecules. In both molecules, the Schiff base ligand, *N'*-[1-(5-chloro-2-oxidophenyl)ethylidene-3-hydroxy-2-naphthohydrazone] forms a tridentate dianion which coordinated to the dibutyltin fragment in a distorted *cis*-C_2_NO_2_Sn trigonal bipyramidal configuration; the axial O—Sn—O angle are 153.03 (13)*^o^* and 152.41 (13)*^o^*.

### Experimental

The Schiff base ligand was prepared by the condensation reaction of 3-hydroxy-2-naphthoic hydrazide with 5-chloro-2-hydroxyacetophenone. The prepared Schiff base (0.74 g, 2.0 mmol), dibutyltin dichloride (0.61 g, 2 mmol) and triethylamine (0.6 ml) were refluxed in 50 ml of ethanol for 5 h. The solution was left for crystallizaton at room temperature during which yellow crystals were obtained.

### Refinement

Hydrogen atoms were placed at calculated positions (C–H 0.95 to 0.98 Å) and were treated as riding on their parent carbon atoms, with *U*(H) set to 1.2–1.5 times *U*_eq_(C). The hydroxy-H was refined with a restraint of 0.84 ± 0.01 Å.

### Crystal data

**Table:**

[Sn(C_4_H_9_)_2_(C_19_H_13_ClN_2_O_3_)]	*F*(000) = 2384
*M**_r_* = 585.67	*D*_x_ = 1.544 Mg m^−^^3^
Monoclinic, *P*2_1_/*n*	Mo *K*α radiation, λ = 0.71073 Å
Hall symbol: -P 2yn	Cell parameters from 9440 reflections
*a* = 24.8256 (13) Å	θ = 3.0–28.3°
*b* = 7.1994 (4) Å	µ = 1.15 mm^−^^1^
*c* = 28.3649 (15) Å	*T* = 100 K
β = 96.376 (1)°	Prism, yellow
*V* = 5038.3 (5) Å^3^	0.25 × 0.25 × 0.15 mm
*Z* = 8	

### Data collection

**Table:**

Bruker APEXII CCD area-detector diffractometer	8881 independent reflections
Radiation source: fine-focus sealed tube	8369 reflections with *I* > 2σ(*I*)
graphite	*R*_int_ = 0.032
ω scans	θ_max_ = 25.0°, θ_min_ = 1.0°
Absorption correction: multi-scan (*SADABS*; Bruker, 2009)	*h* = −29→29
*T*_min_ = 0.762, *T*_max_ = 0.846	*k* = −8→8
37459 measured reflections	*l* = −33→33

### Refinement

**Table:**

Refinement on *F*^2^	Primary atom site location: structure-invariant direct methods
Least-squares matrix: full	Secondary atom site location: difference Fourier map
*R*[*F*^2^ > 2σ(*F*^2^)] = 0.050	Hydrogen site location: inferred from neighbouring sites
*wR*(*F*^2^) = 0.101	H-atom parameters constrained
*S* = 1.32	*w* = 1/[σ^2^(*F*_o_^2^) + (0.0158*P*)^2^ + 27.1939*P*] where *P* = (*F*_o_^2^ + 2*F*_c_^2^)/3
8881 reflections	(Δ/σ)_max_ = 0.002
621 parameters	Δρ_max_ = 0.82 e Å^−^^3^
0 restraints	Δρ_min_ = −1.50 e Å^−^^3^

### Special details

**Table:**

Geometry. All e.s.d.'s (except the e.s.d. in the dihedral angle between two l.s. planes)are estimated using the full covariance matrix. The cell e.s.d.'s are takeninto account individually in the estimation of e.s.d.'s in distances, anglesand torsion angles; correlations between e.s.d.'s in cell parameters are onlyused when they are defined by crystal symmetry. An approximate (isotropic)treatment of cell e.s.d.'s is used for estimating e.s.d.'s involving l.s. planes.
Refinement. Refinement of *F*^2^ against ALL reflections. The weighted *R*-factor *wR* andgoodness of fit *S* are based on *F*^2^, conventional *R*-factors *R* are basedon *F*, with *F* set to zero for negative *F*^2^. The threshold expression of*F*^2^ > σ(*F*^2^) is used only for calculating *R*-factors(gt) *etc*. and isnot relevant to the choice of reflections for refinement. *R*-factors basedon *F*^2^ are statistically about twice as large as those based on *F*, and *R*-factors based on ALL data will be even larger.

### Fractional atomic coordinates and isotropic or equivalent isotropic displacement parameters (Å^2^)

**Table:**

	*x*	*y*	*z*	*U*_iso_*/*U*_eq_	
Sn1	0.339899 (13)	0.57545 (4)	0.642199 (11)	0.01339 (9)	
Sn2	0.663321 (13)	0.45272 (4)	0.866500 (11)	0.01362 (9)	
Cl1	0.09889 (5)	0.79045 (19)	0.48251 (5)	0.0265 (3)	
Cl2	0.90500 (5)	0.15766 (18)	1.01382 (4)	0.0222 (3)	
N1	0.26021 (16)	0.6163 (5)	0.66655 (13)	0.0155 (9)	
N2	0.26107 (16)	0.6250 (5)	0.71576 (13)	0.0156 (9)	
N3	0.73912 (16)	0.3783 (5)	0.83779 (13)	0.0143 (8)	
N4	0.73331 (17)	0.3608 (5)	0.78836 (13)	0.0154 (9)	
O1	0.29315 (13)	0.4587 (5)	0.58494 (11)	0.0181 (7)	
O2	0.35201 (13)	0.6827 (5)	0.71334 (11)	0.0169 (7)	
O3	0.22477 (14)	0.5987 (6)	0.79810 (12)	0.0249 (8)	
H3A	0.2236	0.5980	0.7684	0.037*	
O4	0.71848 (14)	0.5613 (5)	0.91961 (11)	0.0190 (7)	
O5	0.64255 (13)	0.3284 (5)	0.79779 (11)	0.0172 (7)	
O6	0.76138 (14)	0.3611 (6)	0.70211 (12)	0.0240 (8)	
H6A	0.7662	0.3611	0.7319	0.036*	
C1	0.2097 (2)	0.6282 (6)	0.58896 (17)	0.0161 (10)	
C2	0.24893 (19)	0.5410 (6)	0.56375 (17)	0.0158 (10)	
C3	0.2405 (2)	0.5362 (7)	0.51393 (17)	0.0180 (10)	
H3	0.2668	0.4781	0.4970	0.022*	
C4	0.1953 (2)	0.6129 (7)	0.48898 (17)	0.0175 (10)	
H4	0.1905	0.6096	0.4553	0.021*	
C5	0.1570 (2)	0.6953 (7)	0.51414 (18)	0.0191 (11)	
C6	0.1634 (2)	0.7064 (7)	0.56248 (17)	0.0185 (11)	
H6	0.1367	0.7670	0.5785	0.022*	
C7	0.2130 (2)	0.6357 (6)	0.64094 (17)	0.0157 (10)	
C8	0.3104 (2)	0.6565 (6)	0.73630 (16)	0.0144 (10)	
C9	0.3190 (2)	0.6643 (6)	0.78861 (17)	0.0157 (10)	
C10	0.27594 (19)	0.6359 (7)	0.81743 (17)	0.0167 (10)	
C11	0.2865 (2)	0.6438 (7)	0.86580 (17)	0.0188 (11)	
H11	0.2580	0.6214	0.8847	0.023*	
C12	0.3386 (2)	0.6845 (7)	0.88818 (17)	0.0174 (10)	
C13	0.3501 (2)	0.7017 (7)	0.93840 (18)	0.0221 (11)	
H13	0.3221	0.6813	0.9581	0.027*	
C14	0.4011 (2)	0.7474 (7)	0.95847 (18)	0.0253 (12)	
H14	0.4079	0.7604	0.9919	0.030*	
C15	0.4434 (2)	0.7753 (8)	0.93039 (18)	0.0258 (12)	
H15	0.4786	0.8063	0.9450	0.031*	
C16	0.4345 (2)	0.7584 (7)	0.88228 (18)	0.0231 (11)	
H16	0.4636	0.7767	0.8636	0.028*	
C17	0.3820 (2)	0.7133 (6)	0.85986 (17)	0.0164 (10)	
C18	0.3703 (2)	0.7002 (6)	0.81034 (17)	0.0164 (10)	
H18	0.3989	0.7169	0.7911	0.020*	
C19	0.1629 (2)	0.6682 (7)	0.66458 (18)	0.0198 (11)	
H19A	0.1657	0.6011	0.6948	0.030*	
H19B	0.1313	0.6234	0.6440	0.030*	
H19C	0.1589	0.8014	0.6704	0.030*	
C20	0.3635 (2)	0.8241 (7)	0.60966 (17)	0.0181 (10)	
H20A	0.3316	0.8751	0.5898	0.022*	
H20B	0.3745	0.9162	0.6348	0.022*	
C21	0.4097 (2)	0.8011 (7)	0.57894 (17)	0.0196 (11)	
H21A	0.3985	0.7128	0.5529	0.024*	
H21B	0.4172	0.9222	0.5645	0.024*	
C22	0.4611 (2)	0.7306 (8)	0.60696 (19)	0.0261 (12)	
H22A	0.4533	0.6115	0.6222	0.031*	
H22B	0.4729	0.8208	0.6324	0.031*	
C23	0.5069 (2)	0.7017 (8)	0.5766 (2)	0.0341 (14)	
H23A	0.5391	0.6569	0.5965	0.051*	
H23B	0.5153	0.8196	0.5618	0.051*	
H23C	0.4960	0.6098	0.5519	0.051*	
C24	0.3781 (2)	0.3161 (6)	0.66053 (16)	0.0158 (10)	
H24A	0.3497	0.2197	0.6606	0.019*	
H24B	0.4013	0.2827	0.6356	0.019*	
C25	0.4127 (2)	0.3118 (7)	0.70831 (18)	0.0218 (11)	
H25A	0.3916	0.3649	0.7327	0.026*	
H25B	0.4449	0.3916	0.7066	0.026*	
C26	0.4315 (2)	0.1172 (7)	0.72372 (18)	0.0233 (12)	
H26A	0.4526	0.0628	0.6995	0.028*	
H26B	0.3996	0.0371	0.7263	0.028*	
C27	0.4666 (2)	0.1227 (9)	0.7715 (2)	0.0330 (14)	
H27A	0.4744	−0.0044	0.7826	0.050*	
H27B	0.4472	0.1888	0.7947	0.050*	
H27C	0.5007	0.1873	0.7679	0.050*	
C28	0.79522 (19)	0.3650 (6)	0.91222 (16)	0.0143 (10)	
C29	0.76097 (18)	0.4648 (6)	0.93933 (16)	0.0133 (9)	
C30	0.7725 (2)	0.4687 (7)	0.98883 (17)	0.0168 (10)	
H30	0.7496	0.5376	1.0071	0.020*	
C31	0.8163 (2)	0.3747 (7)	1.01155 (17)	0.0167 (10)	
H31	0.8234	0.3775	1.0451	0.020*	
C32	0.84958 (19)	0.2763 (6)	0.98459 (17)	0.0150 (10)	
C33	0.84028 (19)	0.2696 (6)	0.93631 (17)	0.0138 (10)	
H33	0.8640	0.2010	0.9188	0.017*	
C34	0.7875 (2)	0.3568 (6)	0.86017 (17)	0.0156 (10)	
C35	0.68199 (19)	0.3368 (6)	0.77180 (17)	0.0153 (10)	
C36	0.66883 (19)	0.3165 (6)	0.71995 (16)	0.0148 (10)	
C37	0.70804 (19)	0.3304 (7)	0.68733 (17)	0.0165 (10)	
C38	0.69298 (19)	0.3140 (7)	0.63940 (17)	0.0172 (10)	
H38	0.7196	0.3257	0.6180	0.021*	
C39	0.6383 (2)	0.2799 (6)	0.62151 (17)	0.0159 (10)	
C40	0.6216 (2)	0.2574 (7)	0.57239 (18)	0.0221 (11)	
H40	0.6475	0.2685	0.5503	0.027*	
C41	0.5690 (2)	0.2201 (7)	0.55652 (18)	0.0236 (12)	
H41	0.5587	0.2024	0.5236	0.028*	
C42	0.5290 (2)	0.2073 (7)	0.58903 (18)	0.0237 (12)	
H42	0.4923	0.1828	0.5776	0.028*	
C43	0.5438 (2)	0.2303 (7)	0.63622 (18)	0.0223 (11)	
H43	0.5172	0.2222	0.6577	0.027*	
C44	0.5990 (2)	0.2664 (6)	0.65380 (17)	0.0164 (10)	
C45	0.6156 (2)	0.2860 (7)	0.70265 (17)	0.0169 (10)	
H45	0.5892	0.2777	0.7244	0.020*	
C46	0.8346 (2)	0.3182 (7)	0.83287 (17)	0.0183 (11)	
H46A	0.8328	0.4006	0.8052	0.028*	
H46B	0.8332	0.1886	0.8222	0.028*	
H46C	0.8685	0.3401	0.8533	0.028*	
C47	0.6361 (2)	0.2234 (7)	0.90457 (18)	0.0217 (11)	
H47A	0.6578	0.1134	0.8975	0.026*	
H47B	0.6443	0.2498	0.9389	0.026*	
C48	0.5764 (2)	0.1718 (7)	0.89511 (19)	0.0246 (12)	
H48A	0.5690	0.0651	0.9154	0.029*	
H48B	0.5684	0.1324	0.8616	0.029*	
C49	0.5389 (2)	0.3319 (7)	0.90469 (19)	0.0229 (11)	
H49A	0.5518	0.3891	0.9356	0.028*	
H49B	0.5404	0.4277	0.8798	0.028*	
C50	0.4805 (2)	0.2693 (8)	0.9054 (2)	0.0328 (14)	
H50A	0.4576	0.3775	0.9097	0.049*	
H50B	0.4783	0.1822	0.9316	0.049*	
H50C	0.4680	0.2079	0.8753	0.049*	
C51	0.6336 (2)	0.7244 (7)	0.84806 (17)	0.0185 (11)	
H51A	0.6049	0.7546	0.8684	0.022*	
H51B	0.6636	0.8138	0.8560	0.022*	
C52	0.61069 (19)	0.7563 (7)	0.79624 (17)	0.0182 (11)	
H52A	0.6091	0.8918	0.7902	0.022*	
H52B	0.6361	0.7021	0.7755	0.022*	
C53	0.5552 (2)	0.6757 (7)	0.78225 (18)	0.0220 (11)	
H53A	0.5294	0.7298	0.8027	0.026*	
H53B	0.5564	0.5399	0.7878	0.026*	
C54	0.5348 (2)	0.7132 (8)	0.73038 (19)	0.0292 (13)	
H54A	0.4967	0.6758	0.7242	0.044*	
H54B	0.5565	0.6418	0.7099	0.044*	
H54C	0.5381	0.8460	0.7237	0.044*	

### Atomic displacement parameters (Å^2^)

**Table:**

	*U*^11^	*U*^22^	*U*^33^	*U*^12^	*U*^13^	*U*^23^
Sn1	0.01504 (18)	0.01007 (16)	0.01526 (16)	0.00157 (13)	0.00262 (12)	0.00071 (12)
Sn2	0.01455 (18)	0.01240 (16)	0.01387 (16)	0.00251 (13)	0.00139 (12)	−0.00070 (13)
Cl1	0.0267 (7)	0.0260 (7)	0.0248 (7)	0.0067 (6)	−0.0070 (5)	0.0001 (5)
Cl2	0.0198 (6)	0.0236 (6)	0.0222 (6)	0.0062 (5)	−0.0030 (5)	0.0017 (5)
N1	0.020 (2)	0.012 (2)	0.015 (2)	0.0007 (17)	0.0031 (17)	−0.0005 (16)
N2	0.018 (2)	0.014 (2)	0.014 (2)	0.0022 (17)	0.0020 (16)	0.0017 (16)
N3	0.018 (2)	0.0119 (19)	0.0124 (19)	0.0017 (16)	0.0003 (16)	−0.0001 (15)
N4	0.021 (2)	0.012 (2)	0.013 (2)	0.0044 (17)	0.0009 (16)	−0.0008 (16)
O1	0.0158 (18)	0.0165 (17)	0.0218 (17)	0.0034 (14)	0.0014 (14)	−0.0027 (14)
O2	0.0186 (18)	0.0145 (17)	0.0179 (17)	0.0014 (14)	0.0039 (14)	−0.0009 (14)
O3	0.0180 (19)	0.037 (2)	0.0202 (18)	−0.0022 (17)	0.0053 (15)	−0.0045 (17)
O4	0.0200 (18)	0.0168 (17)	0.0195 (17)	0.0036 (15)	−0.0007 (14)	−0.0050 (14)
O5	0.0149 (18)	0.0212 (18)	0.0158 (16)	−0.0008 (14)	0.0034 (14)	−0.0036 (14)
O6	0.0142 (19)	0.038 (2)	0.0196 (18)	0.0012 (16)	0.0028 (14)	−0.0021 (17)
C1	0.018 (3)	0.008 (2)	0.022 (2)	−0.0023 (19)	0.001 (2)	0.0013 (19)
C2	0.015 (2)	0.009 (2)	0.023 (2)	0.0003 (19)	0.0003 (19)	0.001 (2)
C3	0.023 (3)	0.010 (2)	0.021 (2)	0.000 (2)	0.004 (2)	−0.001 (2)
C4	0.022 (3)	0.014 (2)	0.016 (2)	−0.002 (2)	0.000 (2)	−0.0022 (19)
C5	0.021 (3)	0.012 (2)	0.023 (3)	0.000 (2)	−0.003 (2)	0.001 (2)
C6	0.021 (3)	0.011 (2)	0.023 (3)	0.001 (2)	0.002 (2)	−0.002 (2)
C7	0.021 (3)	0.004 (2)	0.022 (3)	−0.0004 (19)	0.003 (2)	−0.0012 (18)
C8	0.020 (3)	0.008 (2)	0.016 (2)	0.0071 (19)	0.005 (2)	−0.0004 (18)
C9	0.018 (3)	0.009 (2)	0.021 (2)	0.0017 (19)	0.003 (2)	0.0001 (19)
C10	0.011 (2)	0.015 (2)	0.025 (3)	0.0046 (19)	0.007 (2)	−0.001 (2)
C11	0.017 (3)	0.017 (3)	0.024 (3)	0.004 (2)	0.010 (2)	0.002 (2)
C12	0.022 (3)	0.011 (2)	0.021 (2)	0.005 (2)	0.005 (2)	0.0002 (19)
C13	0.031 (3)	0.014 (2)	0.022 (3)	0.003 (2)	0.006 (2)	0.002 (2)
C14	0.042 (3)	0.018 (3)	0.015 (2)	0.007 (2)	0.000 (2)	0.000 (2)
C15	0.029 (3)	0.023 (3)	0.023 (3)	0.000 (2)	−0.006 (2)	0.001 (2)
C16	0.025 (3)	0.022 (3)	0.022 (3)	0.000 (2)	0.002 (2)	0.002 (2)
C17	0.020 (3)	0.010 (2)	0.020 (2)	0.004 (2)	0.003 (2)	0.0007 (19)
C18	0.019 (3)	0.011 (2)	0.020 (2)	0.003 (2)	0.007 (2)	−0.0010 (19)
C19	0.020 (3)	0.016 (2)	0.023 (3)	0.000 (2)	0.002 (2)	0.000 (2)
C20	0.019 (3)	0.013 (2)	0.023 (3)	0.004 (2)	0.006 (2)	0.002 (2)
C21	0.023 (3)	0.017 (2)	0.019 (2)	−0.002 (2)	0.003 (2)	0.002 (2)
C22	0.027 (3)	0.022 (3)	0.029 (3)	−0.002 (2)	0.004 (2)	0.007 (2)
C23	0.029 (3)	0.017 (3)	0.058 (4)	−0.002 (2)	0.011 (3)	0.004 (3)
C24	0.020 (3)	0.009 (2)	0.019 (2)	0.006 (2)	0.004 (2)	0.0058 (19)
C25	0.018 (3)	0.023 (3)	0.024 (3)	0.006 (2)	0.001 (2)	0.004 (2)
C26	0.022 (3)	0.025 (3)	0.022 (3)	0.008 (2)	0.004 (2)	0.007 (2)
C27	0.036 (3)	0.034 (3)	0.029 (3)	0.014 (3)	0.003 (3)	0.009 (3)
C28	0.015 (2)	0.011 (2)	0.017 (2)	−0.0028 (19)	0.0004 (19)	−0.0012 (18)
C29	0.009 (2)	0.012 (2)	0.019 (2)	−0.0032 (19)	0.0009 (18)	−0.0004 (19)
C30	0.017 (3)	0.013 (2)	0.021 (2)	−0.004 (2)	0.006 (2)	−0.004 (2)
C31	0.018 (3)	0.013 (2)	0.019 (2)	−0.003 (2)	0.001 (2)	−0.0006 (19)
C32	0.010 (2)	0.012 (2)	0.022 (3)	0.0022 (19)	−0.0038 (19)	−0.0013 (19)
C33	0.011 (2)	0.009 (2)	0.022 (2)	−0.0018 (18)	0.0026 (19)	−0.0027 (19)
C34	0.019 (3)	0.009 (2)	0.019 (2)	−0.0020 (19)	0.002 (2)	−0.0008 (19)
C35	0.014 (3)	0.010 (2)	0.022 (2)	0.0049 (19)	0.002 (2)	−0.0015 (19)
C36	0.017 (3)	0.010 (2)	0.017 (2)	0.0046 (19)	0.0026 (19)	0.0000 (19)
C37	0.010 (2)	0.014 (2)	0.025 (3)	0.0009 (19)	0.004 (2)	−0.003 (2)
C38	0.013 (2)	0.021 (3)	0.019 (2)	0.004 (2)	0.0044 (19)	0.000 (2)
C39	0.017 (3)	0.012 (2)	0.018 (2)	0.007 (2)	0.0003 (19)	0.0004 (19)
C40	0.029 (3)	0.017 (3)	0.020 (3)	0.005 (2)	0.000 (2)	0.000 (2)
C41	0.030 (3)	0.020 (3)	0.019 (3)	0.003 (2)	−0.004 (2)	−0.003 (2)
C42	0.025 (3)	0.018 (3)	0.026 (3)	−0.003 (2)	−0.006 (2)	0.002 (2)
C43	0.023 (3)	0.020 (3)	0.024 (3)	−0.002 (2)	0.002 (2)	0.000 (2)
C44	0.017 (3)	0.012 (2)	0.020 (2)	0.003 (2)	−0.001 (2)	0.0004 (19)
C45	0.016 (3)	0.014 (2)	0.021 (2)	0.005 (2)	0.003 (2)	−0.001 (2)
C46	0.023 (3)	0.015 (2)	0.017 (2)	0.001 (2)	0.001 (2)	−0.001 (2)
C47	0.027 (3)	0.015 (2)	0.024 (3)	0.002 (2)	0.006 (2)	0.004 (2)
C48	0.034 (3)	0.017 (3)	0.024 (3)	−0.007 (2)	0.008 (2)	0.000 (2)
C49	0.023 (3)	0.017 (3)	0.029 (3)	0.002 (2)	−0.001 (2)	0.004 (2)
C50	0.023 (3)	0.025 (3)	0.052 (4)	−0.006 (2)	0.011 (3)	−0.007 (3)
C51	0.019 (3)	0.019 (3)	0.018 (2)	0.003 (2)	0.005 (2)	0.001 (2)
C52	0.011 (2)	0.020 (3)	0.024 (3)	0.004 (2)	0.003 (2)	0.004 (2)
C53	0.013 (3)	0.021 (3)	0.032 (3)	0.003 (2)	0.002 (2)	0.005 (2)
C54	0.030 (3)	0.027 (3)	0.029 (3)	0.001 (3)	−0.007 (2)	0.000 (2)

### Geometric parameters (Å, °)

**Table:**

Sn1—O1	2.067 (3)	C23—H23C	0.9800
Sn1—C20	2.126 (5)	C24—C25	1.522 (7)
Sn1—C24	2.132 (4)	C24—H24A	0.9900
Sn1—O2	2.150 (3)	C24—H24B	0.9900
Sn1—N1	2.186 (4)	C25—C26	1.526 (7)
Sn2—O4	2.073 (3)	C25—H25A	0.9900
Sn2—C47	2.123 (5)	C25—H25B	0.9900
Sn2—C51	2.135 (5)	C26—C27	1.528 (7)
Sn2—O5	2.155 (3)	C26—H26A	0.9900
Sn2—N3	2.198 (4)	C26—H26B	0.9900
Cl1—C5	1.751 (5)	C27—H27A	0.9800
Cl2—C32	1.748 (5)	C27—H27B	0.9800
N1—C7	1.316 (6)	C27—H27C	0.9800
N1—N2	1.395 (5)	C28—C29	1.406 (7)
N2—C8	1.316 (6)	C28—C33	1.421 (7)
N3—C34	1.304 (6)	C28—C34	1.469 (6)
N3—N4	1.399 (5)	C29—C30	1.402 (6)
N4—C35	1.319 (6)	C30—C31	1.378 (7)
O1—C2	1.331 (6)	C30—H30	0.9500
O2—C8	1.294 (6)	C31—C32	1.383 (7)
O3—C10	1.353 (6)	C31—H31	0.9500
O3—H3A	0.8400	C32—C33	1.364 (7)
O4—C29	1.333 (6)	C33—H33	0.9500
O5—C35	1.291 (6)	C34—C46	1.498 (7)
O6—C37	1.362 (6)	C35—C36	1.478 (6)
O6—H6A	0.8400	C36—C45	1.375 (7)
C1—C2	1.417 (7)	C36—C37	1.419 (7)
C1—C6	1.418 (7)	C37—C38	1.375 (7)
C1—C7	1.469 (7)	C38—C39	1.418 (7)
C2—C3	1.406 (7)	C38—H38	0.9500
C3—C4	1.374 (7)	C39—C44	1.414 (7)
C3—H3	0.9500	C39—C40	1.418 (7)
C4—C5	1.385 (7)	C40—C41	1.359 (8)
C4—H4	0.9500	C40—H40	0.9500
C5—C6	1.365 (7)	C41—C42	1.431 (8)
C6—H6	0.9500	C41—H41	0.9500
C7—C19	1.496 (7)	C42—C43	1.358 (7)
C8—C9	1.476 (6)	C42—H42	0.9500
C9—C18	1.377 (7)	C43—C44	1.427 (7)
C9—C10	1.431 (7)	C43—H43	0.9500
C10—C11	1.369 (7)	C44—C45	1.408 (7)
C11—C12	1.407 (7)	C45—H45	0.9500
C11—H11	0.9500	C46—H46A	0.9800
C12—C13	1.427 (7)	C46—H46B	0.9800
C12—C17	1.427 (7)	C46—H46C	0.9800
C13—C14	1.370 (8)	C47—C48	1.524 (7)
C13—H13	0.9500	C47—H47A	0.9900
C14—C15	1.402 (8)	C47—H47B	0.9900
C14—H14	0.9500	C48—C49	1.525 (7)
C15—C16	1.363 (7)	C48—H48A	0.9900
C15—H15	0.9500	C48—H48B	0.9900
C16—C17	1.424 (7)	C49—C50	1.519 (7)
C16—H16	0.9500	C49—H49A	0.9900
C17—C18	1.405 (7)	C49—H49B	0.9900
C18—H18	0.9500	C50—H50A	0.9800
C19—H19A	0.9800	C50—H50B	0.9800
C19—H19B	0.9800	C50—H50C	0.9800
C19—H19C	0.9800	C51—C52	1.533 (7)
C20—C21	1.525 (7)	C51—H51A	0.9900
C20—H20A	0.9900	C51—H51B	0.9900
C20—H20B	0.9900	C52—C53	1.507 (7)
C21—C22	1.514 (7)	C52—H52A	0.9900
C21—H21A	0.9900	C52—H52B	0.9900
C21—H21B	0.9900	C53—C54	1.526 (7)
C22—C23	1.514 (8)	C53—H53A	0.9900
C22—H22A	0.9900	C53—H53B	0.9900
C22—H22B	0.9900	C54—H54A	0.9800
C23—H23A	0.9800	C54—H54B	0.9800
C23—H23B	0.9800	C54—H54C	0.9800
			
O1—Sn1—C20	99.15 (17)	C24—C25—H25A	108.9
O1—Sn1—C24	91.79 (16)	C26—C25—H25A	108.9
C20—Sn1—C24	134.95 (19)	C24—C25—H25B	108.9
O1—Sn1—O2	153.02 (13)	C26—C25—H25B	108.9
C20—Sn1—O2	95.08 (16)	H25A—C25—H25B	107.7
C24—Sn1—O2	94.22 (15)	C25—C26—C27	110.9 (5)
O1—Sn1—N1	81.61 (14)	C25—C26—H26A	109.5
C20—Sn1—N1	109.07 (16)	C27—C26—H26A	109.5
C24—Sn1—N1	115.74 (17)	C25—C26—H26B	109.5
O2—Sn1—N1	72.08 (13)	C27—C26—H26B	109.5
O4—Sn2—C47	98.34 (17)	H26A—C26—H26B	108.1
O4—Sn2—C51	90.85 (17)	C26—C27—H27A	109.5
C47—Sn2—C51	135.8 (2)	C26—C27—H27B	109.5
O4—Sn2—O5	152.42 (13)	H27A—C27—H27B	109.5
C47—Sn2—O5	94.55 (17)	C26—C27—H27C	109.5
C51—Sn2—O5	96.75 (16)	H27A—C27—H27C	109.5
O4—Sn2—N3	80.65 (14)	H27B—C27—H27C	109.5
C47—Sn2—N3	109.41 (17)	C29—C28—C33	118.4 (4)
C51—Sn2—N3	114.78 (17)	C29—C28—C34	123.4 (4)
O5—Sn2—N3	72.08 (13)	C33—C28—C34	118.2 (4)
C7—N1—N2	117.5 (4)	O4—C29—C30	118.3 (4)
C7—N1—Sn1	128.4 (3)	O4—C29—C28	122.4 (4)
N2—N1—Sn1	114.1 (3)	C30—C29—C28	119.3 (4)
C8—N2—N1	111.1 (4)	C31—C30—C29	121.4 (4)
C34—N3—N4	117.7 (4)	C31—C30—H30	119.3
C34—N3—Sn2	129.0 (3)	C29—C30—H30	119.3
N4—N3—Sn2	113.3 (3)	C30—C31—C32	118.8 (4)
C35—N4—N3	110.9 (4)	C30—C31—H31	120.6
C2—O1—Sn1	122.5 (3)	C32—C31—H31	120.6
C8—O2—Sn1	112.6 (3)	C33—C32—C31	121.9 (4)
C10—O3—H3A	109.5	C33—C32—Cl2	119.7 (4)
C29—O4—Sn2	122.5 (3)	C31—C32—Cl2	118.4 (4)
C35—O5—Sn2	112.1 (3)	C32—C33—C28	120.1 (4)
C37—O6—H6A	109.5	C32—C33—H33	119.9
C2—C1—C6	118.1 (4)	C28—C33—H33	119.9
C2—C1—C7	123.9 (4)	N3—C34—C28	119.7 (4)
C6—C1—C7	118.0 (4)	N3—C34—C46	120.0 (4)
O1—C2—C3	117.8 (4)	C28—C34—C46	120.3 (4)
O1—C2—C1	123.2 (4)	O5—C35—N4	124.5 (4)
C3—C2—C1	118.9 (4)	O5—C35—C36	117.8 (4)
C4—C3—C2	122.0 (5)	N4—C35—C36	117.7 (4)
C4—C3—H3	119.0	C45—C36—C37	118.7 (4)
C2—C3—H3	119.0	C45—C36—C35	117.9 (4)
C3—C4—C5	118.4 (5)	C37—C36—C35	123.4 (4)
C3—C4—H4	120.8	O6—C37—C38	117.8 (4)
C5—C4—H4	120.8	O6—C37—C36	121.6 (4)
C6—C5—C4	122.1 (5)	C38—C37—C36	120.5 (4)
C6—C5—Cl1	119.3 (4)	C37—C38—C39	120.9 (4)
C4—C5—Cl1	118.5 (4)	C37—C38—H38	119.6
C5—C6—C1	120.5 (5)	C39—C38—H38	119.6
C5—C6—H6	119.8	C44—C39—C38	118.9 (4)
C1—C6—H6	119.8	C44—C39—C40	118.8 (5)
N1—C7—C1	119.9 (4)	C38—C39—C40	122.4 (5)
N1—C7—C19	120.2 (4)	C41—C40—C39	120.9 (5)
C1—C7—C19	119.9 (4)	C41—C40—H40	119.6
O2—C8—N2	123.9 (4)	C39—C40—H40	119.6
O2—C8—C9	117.8 (4)	C40—C41—C42	120.6 (5)
N2—C8—C9	118.3 (4)	C40—C41—H41	119.7
C18—C9—C10	118.9 (4)	C42—C41—H41	119.7
C18—C9—C8	118.7 (4)	C43—C42—C41	119.8 (5)
C10—C9—C8	122.4 (4)	C43—C42—H42	120.1
O3—C10—C11	118.8 (4)	C41—C42—H42	120.1
O3—C10—C9	121.6 (4)	C42—C43—C44	120.7 (5)
C11—C10—C9	119.6 (5)	C42—C43—H43	119.7
C10—C11—C12	121.7 (5)	C44—C43—H43	119.7
C10—C11—H11	119.2	C45—C44—C39	118.8 (4)
C12—C11—H11	119.2	C45—C44—C43	121.8 (5)
C11—C12—C13	122.6 (5)	C39—C44—C43	119.3 (4)
C11—C12—C17	119.3 (4)	C36—C45—C44	122.2 (5)
C13—C12—C17	118.1 (5)	C36—C45—H45	118.9
C14—C13—C12	120.5 (5)	C44—C45—H45	118.9
C14—C13—H13	119.7	C34—C46—H46A	109.5
C12—C13—H13	119.7	C34—C46—H46B	109.5
C13—C14—C15	121.0 (5)	H46A—C46—H46B	109.5
C13—C14—H14	119.5	C34—C46—H46C	109.5
C15—C14—H14	119.5	H46A—C46—H46C	109.5
C16—C15—C14	120.5 (5)	H46B—C46—H46C	109.5
C16—C15—H15	119.7	C48—C47—Sn2	117.3 (3)
C14—C15—H15	119.7	C48—C47—H47A	108.0
C15—C16—C17	120.4 (5)	Sn2—C47—H47A	108.0
C15—C16—H16	119.8	C48—C47—H47B	108.0
C17—C16—H16	119.8	Sn2—C47—H47B	108.0
C18—C17—C16	122.6 (5)	H47A—C47—H47B	107.2
C18—C17—C12	117.9 (5)	C47—C48—C49	112.7 (4)
C16—C17—C12	119.5 (5)	C47—C48—H48A	109.0
C9—C18—C17	122.5 (5)	C49—C48—H48A	109.0
C9—C18—H18	118.7	C47—C48—H48B	109.0
C17—C18—H18	118.7	C49—C48—H48B	109.0
C7—C19—H19A	109.5	H48A—C48—H48B	107.8
C7—C19—H19B	109.5	C50—C49—C48	112.4 (4)
H19A—C19—H19B	109.5	C50—C49—H49A	109.1
C7—C19—H19C	109.5	C48—C49—H49A	109.1
H19A—C19—H19C	109.5	C50—C49—H49B	109.1
H19B—C19—H19C	109.5	C48—C49—H49B	109.1
C21—C20—Sn1	114.7 (3)	H49A—C49—H49B	107.9
C21—C20—H20A	108.6	C49—C50—H50A	109.5
Sn1—C20—H20A	108.6	C49—C50—H50B	109.5
C21—C20—H20B	108.6	H50A—C50—H50B	109.5
Sn1—C20—H20B	108.6	C49—C50—H50C	109.5
H20A—C20—H20B	107.6	H50A—C50—H50C	109.5
C22—C21—C20	112.4 (4)	H50B—C50—H50C	109.5
C22—C21—H21A	109.1	C52—C51—Sn2	117.0 (3)
C20—C21—H21A	109.1	C52—C51—H51A	108.1
C22—C21—H21B	109.1	Sn2—C51—H51A	108.1
C20—C21—H21B	109.1	C52—C51—H51B	108.1
H21A—C21—H21B	107.9	Sn2—C51—H51B	108.1
C21—C22—C23	112.9 (5)	H51A—C51—H51B	107.3
C21—C22—H22A	109.0	C53—C52—C51	115.2 (4)
C23—C22—H22A	109.0	C53—C52—H52A	108.5
C21—C22—H22B	109.0	C51—C52—H52A	108.5
C23—C22—H22B	109.0	C53—C52—H52B	108.5
H22A—C22—H22B	107.8	C51—C52—H52B	108.5
C22—C23—H23A	109.5	H52A—C52—H52B	107.5
C22—C23—H23B	109.5	C52—C53—C54	112.4 (4)
H23A—C23—H23B	109.5	C52—C53—H53A	109.1
C22—C23—H23C	109.5	C54—C53—H53A	109.1
H23A—C23—H23C	109.5	C52—C53—H53B	109.1
H23B—C23—H23C	109.5	C54—C53—H53B	109.1
C25—C24—Sn1	115.3 (3)	H53A—C53—H53B	107.8
C25—C24—H24A	108.5	C53—C54—H54A	109.5
Sn1—C24—H24A	108.5	C53—C54—H54B	109.5
C25—C24—H24B	108.5	H54A—C54—H54B	109.5
Sn1—C24—H24B	108.5	C53—C54—H54C	109.5
H24A—C24—H24B	107.5	H54A—C54—H54C	109.5
C24—C25—C26	113.5 (4)	H54B—C54—H54C	109.5
			
O1—Sn1—N1—C7	−27.5 (4)	C11—C12—C17—C16	178.7 (5)
C20—Sn1—N1—C7	69.4 (4)	C13—C12—C17—C16	−0.2 (7)
C24—Sn1—N1—C7	−115.4 (4)	C10—C9—C18—C17	−1.1 (7)
O2—Sn1—N1—C7	158.6 (4)	C8—C9—C18—C17	178.9 (4)
O1—Sn1—N1—N2	153.6 (3)	C16—C17—C18—C9	−177.2 (5)
C20—Sn1—N1—N2	−109.6 (3)	C12—C17—C18—C9	1.2 (7)
C24—Sn1—N1—N2	65.7 (3)	O1—Sn1—C20—C21	−79.3 (4)
O2—Sn1—N1—N2	−20.3 (3)	C24—Sn1—C20—C21	22.6 (5)
C7—N1—N2—C8	−162.1 (4)	O2—Sn1—C20—C21	123.7 (4)
Sn1—N1—N2—C8	17.0 (5)	N1—Sn1—C20—C21	−163.5 (3)
O4—Sn2—N3—C34	23.2 (4)	Sn1—C20—C21—C22	−60.6 (5)
C47—Sn2—N3—C34	−72.5 (4)	C20—C21—C22—C23	178.3 (4)
C51—Sn2—N3—C34	109.8 (4)	O1—Sn1—C24—C25	−169.9 (4)
O5—Sn2—N3—C34	−161.0 (4)	C20—Sn1—C24—C25	85.2 (4)
O4—Sn2—N3—N4	−153.8 (3)	O2—Sn1—C24—C25	−16.3 (4)
C47—Sn2—N3—N4	110.6 (3)	N1—Sn1—C24—C25	−88.4 (4)
C51—Sn2—N3—N4	−67.2 (3)	Sn1—C24—C25—C26	170.4 (3)
O5—Sn2—N3—N4	22.1 (3)	C24—C25—C26—C27	179.2 (4)
C34—N3—N4—C35	163.4 (4)	Sn2—O4—C29—C30	−135.1 (4)
Sn2—N3—N4—C35	−19.3 (4)	Sn2—O4—C29—C28	46.9 (6)
C20—Sn1—O1—C2	−61.1 (4)	C33—C28—C29—O4	178.6 (4)
C24—Sn1—O1—C2	162.8 (4)	C34—C28—C29—O4	−0.2 (7)
O2—Sn1—O1—C2	59.9 (5)	C33—C28—C29—C30	0.6 (7)
N1—Sn1—O1—C2	47.0 (4)	C34—C28—C29—C30	−178.2 (4)
O1—Sn1—O2—C8	7.4 (5)	O4—C29—C30—C31	−178.9 (4)
C20—Sn1—O2—C8	129.2 (3)	C28—C29—C30—C31	−0.9 (7)
C24—Sn1—O2—C8	−95.0 (3)	C29—C30—C31—C32	0.6 (7)
N1—Sn1—O2—C8	20.7 (3)	C30—C31—C32—C33	−0.1 (7)
C47—Sn2—O4—C29	60.2 (4)	C30—C31—C32—Cl2	180.0 (4)
C51—Sn2—O4—C29	−163.2 (4)	C31—C32—C33—C28	−0.2 (7)
O5—Sn2—O4—C29	−56.8 (5)	Cl2—C32—C33—C28	179.8 (4)
N3—Sn2—O4—C29	−48.2 (3)	C29—C28—C33—C32	−0.1 (7)
O4—Sn2—O5—C35	−12.8 (5)	C34—C28—C33—C32	178.8 (4)
C47—Sn2—O5—C35	−130.6 (3)	N4—N3—C34—C28	−177.1 (4)
C51—Sn2—O5—C35	92.2 (3)	Sn2—N3—C34—C28	6.1 (6)
N3—Sn2—O5—C35	−21.7 (3)	N4—N3—C34—C46	1.0 (6)
Sn1—O1—C2—C3	138.3 (4)	Sn2—N3—C34—C46	−175.8 (3)
Sn1—O1—C2—C1	−43.3 (6)	C29—C28—C34—N3	−26.8 (7)
C6—C1—C2—O1	−178.1 (4)	C33—C28—C34—N3	154.4 (4)
C7—C1—C2—O1	−0.7 (7)	C29—C28—C34—C46	155.1 (5)
C6—C1—C2—C3	0.2 (7)	C33—C28—C34—C46	−23.7 (6)
C7—C1—C2—C3	177.6 (4)	Sn2—O5—C35—N4	20.6 (6)
O1—C2—C3—C4	178.2 (4)	Sn2—O5—C35—C36	−159.9 (3)
C1—C2—C3—C4	−0.2 (7)	N3—N4—C35—O5	−0.7 (6)
C2—C3—C4—C5	−0.7 (7)	N3—N4—C35—C36	179.8 (4)
C3—C4—C5—C6	1.7 (8)	O5—C35—C36—C45	−1.4 (7)
C3—C4—C5—Cl1	−179.2 (4)	N4—C35—C36—C45	178.1 (4)
C4—C5—C6—C1	−1.8 (8)	O5—C35—C36—C37	177.4 (4)
Cl1—C5—C6—C1	179.1 (4)	N4—C35—C36—C37	−3.1 (7)
C2—C1—C6—C5	0.8 (7)	C45—C36—C37—O6	179.8 (4)
C7—C1—C6—C5	−176.8 (4)	C35—C36—C37—O6	1.0 (7)
N2—N1—C7—C1	179.9 (4)	C45—C36—C37—C38	0.2 (7)
Sn1—N1—C7—C1	0.9 (6)	C35—C36—C37—C38	−178.6 (4)
N2—N1—C7—C19	0.8 (6)	O6—C37—C38—C39	179.3 (4)
Sn1—N1—C7—C19	−178.2 (3)	C36—C37—C38—C39	−1.1 (7)
C2—C1—C7—N1	22.4 (7)	C37—C38—C39—C44	1.2 (7)
C6—C1—C7—N1	−160.2 (4)	C37—C38—C39—C40	−178.3 (5)
C2—C1—C7—C19	−158.6 (5)	C44—C39—C40—C41	−1.2 (7)
C6—C1—C7—C19	18.8 (6)	C38—C39—C40—C41	178.3 (5)
Sn1—O2—C8—N2	−20.6 (6)	C39—C40—C41—C42	1.6 (8)
Sn1—O2—C8—C9	159.9 (3)	C40—C41—C42—C43	−0.8 (8)
N1—N2—C8—O2	2.4 (6)	C41—C42—C43—C44	−0.2 (8)
N1—N2—C8—C9	−178.2 (4)	C38—C39—C44—C45	−0.5 (7)
O2—C8—C9—C18	0.5 (6)	C40—C39—C44—C45	179.0 (4)
N2—C8—C9—C18	−179.0 (4)	C38—C39—C44—C43	−179.4 (5)
O2—C8—C9—C10	−179.5 (4)	C40—C39—C44—C43	0.2 (7)
N2—C8—C9—C10	1.1 (7)	C42—C43—C44—C45	−178.3 (5)
C18—C9—C10—O3	−179.5 (4)	C42—C43—C44—C39	0.6 (7)
C8—C9—C10—O3	0.5 (7)	C37—C36—C45—C44	0.6 (7)
C18—C9—C10—C11	−0.3 (7)	C35—C36—C45—C44	179.4 (4)
C8—C9—C10—C11	179.6 (4)	C39—C44—C45—C36	−0.4 (7)
O3—C10—C11—C12	−179.1 (4)	C43—C44—C45—C36	178.5 (5)
C9—C10—C11—C12	1.7 (7)	O4—Sn2—C47—C48	143.3 (4)
C10—C11—C12—C13	177.1 (5)	C51—Sn2—C47—C48	43.4 (5)
C10—C11—C12—C17	−1.7 (7)	O5—Sn2—C47—C48	−61.2 (4)
C11—C12—C13—C14	−177.9 (5)	N3—Sn2—C47—C48	−133.7 (4)
C17—C12—C13—C14	1.0 (7)	Sn2—C47—C48—C49	−57.6 (5)
C12—C13—C14—C15	−1.0 (8)	C47—C48—C49—C50	−167.9 (5)
C13—C14—C15—C16	0.3 (8)	O4—Sn2—C51—C52	150.9 (4)
C14—C15—C16—C17	0.4 (8)	C47—Sn2—C51—C52	−106.2 (4)
C15—C16—C17—C18	177.9 (5)	O5—Sn2—C51—C52	−2.6 (4)
C15—C16—C17—C12	−0.5 (7)	N3—Sn2—C51—C52	70.8 (4)
C11—C12—C17—C18	0.2 (7)	Sn2—C51—C52—C53	75.6 (5)
C13—C12—C17—C18	−178.6 (4)	C51—C52—C53—C54	179.8 (4)

### Hydrogen-bond geometry (Å, °)

**Table:**

*D*—H···*A*	*D*—H	H···*A*	*D*···*A*	*D*—H···*A*
O3—H3A···N2	0.84	1.85	2.602 (5)	147
O6—H6A···N4	0.84	1.88	2.617 (5)	146
